# Supplementary material for: A glimpse of the paleome in endolithic microbial communities
Source: Microbiome. 2023 Sep 25;11:210. doi: 10.1186/s40168-023-01647-2 (PMC10518947; doi:10.1186/s40168-023-01647-2)
Supplement: Supplementary file 2 — Additional file 1: Supplementary figures: Fig. S1. Pore size distribution of limestone samples with connected pore space (throats >26 µm). LOD = limit of detection. Fig. S2. Pore space characteristics of sample H22-30 determined by µCT analysis. a) Moldic pores (up to large mesopores) dominate over fine fractures in the oolithic packstone. Scale: 0.5 mm. Plug diameter 13 mm. b) Vertical section shows porosity >26 µm. The dashed line marks the position of c. c) Horizontal section shows a moldic pore in a gastropod fossil. d) Reconstructed pore space. Colors mark parts of the pore system connected by throats >26 µm. Fig. S3. Pore space characteristics of sample KS36-H32 determined by µCT analysis. a) The packstone shows minimal alteration (Fe-mineral-stained spots, not visible). Scale: 0.5 mm. Plug diameter 13 mm. b) In the vertical section, connected pores up to ~26 µm (resolution limit) are assumed. The dashed line marks the position of c. c) Horizontal section showing the tight matrix that exhibits no pores connected by throats >26 µm. Fig. S4. Pore space characteristics of sample CM1-H32 determined by µCT analysis. a) The wackestone lacks alteration. Scale: 0.5 mm. Plug diameter 13 mm. b) In the vertical section, connected pores up to ~26 µm (resolution limit) are assumed. The dashed line marks the position of c. c) Horizontal section showing the tight matrix that exhibits no pores connected by throats >26 µm. Fig. S5. Pore space characteristics of sample INF-MB2 determined by µCT analysis. a) The calcareous mudstone lacks alteration. Scale: 0.5 mm. Plug diameter 13 mm. b) In the vertical section, connected pores up to ~26 µm (resolution limit) are assumed. The dashed line marks the position of c. c) Horizontal section showing the tight matrix that exhibits no pores connected by throats >26 µm. Fig. S6. Pore space characteristics of sample INF-MB3 determined by µCT analysis. a) The packstone/grainstone sample lacks alteration. Scale: 0.5 mm. Plug diameter 13 mm. b [file 40168_2023_1647_MOESM1_ESM.pdf]

SUPPLEMENTARY MATERIAL: A glimpse of the paleome in endolithic microbial communities

Carl-Eric Wegner[1], Raphaela Stahl[2], Irina Velsko[2], Alex Hübner[2], Zandra Fagernäs[2], Robert Lehmann[3], Thomas Ritschel[3], Kai U. Totsche[3], Christina Warinner[2,4], and Kirsten Küsel[1,5]

[1]Institute of Biodiversity, Aquatic Geomicrobiology, Friedrich Schiller University, Dornburger Str. 159, 07743 Jena, Germany

[2]Department of Archaeogenetics, Max Planck Institute for Evolutionary Anthropology, Deutscher Platz 6, 04103 Leipzig, Germany

[3]Institute of Geosciences, Hydrogeology, Friedrich Schiller University Jena, Burgweg 11, 07749 Jena, Germany

[4]Department of Anthropology, Harvard University, Cambridge, MA, USA

[5]German Center for Integrative Biodiversity Research (iDiv) Halle-Jena-Leipzig, Puschstraße 4, 04103 Leipzig, Germany

# Corresponding author:

Kirsten Küsel

Email address: kirsten.kuesel@uni-jena.de

SUPPLEMENTARY MATERIAL

FIG S1. Pore size distribution of limestone samples with connected pore space (throats >26 µm). LOD = limit of detection.

FIG S2. Pore space characteristics of sample H22-30 by µCT analysis. a) Moldic pores (up to large mesopores) dominate over fine fractures in the oolithic packstone. Scale: 0.5 mm. Plug diameter 13 mm. b) Vertical section shows porosity >26 µm. The dashed line marks the position of c. c) Horizontal section shows a moldic pore in a gastropod fossil. d) Reconstructed pore space. Colors mark parts of the pore system connected by throats >26 µm.

FIG S3. Pore space characteristics of sample KS36-H32 by µCT analysis. a) The packstone shows minimal alteration (Fe-mineral-stained spots, not visible). Scale: 0.5 mm. Plug diameter 13 mm. b) In the vertical section, connected pores up to ~26 µm (resolution limit) are assumed. The dashed line marks the position of c. c) Horizontal section showing the tight matrix that exhibits no pores connected by throats >26 µm.

FIG S4. Pore space characteristics of sample CM1-H32 by µCT analysis. a) The wackestone lacks alteration. Scale: 0.5 mm. Plug diameter 13 mm. b) In the vertical section, connected pores up to ~26 µm (resolution limit) are assumed. The dashed line marks the position of c. c) Horizontal section showing the tight matrix that exhibits no pores connected by throats >26 µm.

FIG S5. Pore space characteristics of sample INF-MB2 by µCT analysis. a) The calcareous mudstone lacks alteration. Scale: 0.5 mm. Plug diameter 13 mm. b) In the vertical section, connected pores up to ~26 µm (resolution limit) are assumed. The dashed line marks the position of c. c) Horizontal section showing the tight matrix that exhibits no pores connected by throats >26 µm.

FIG S6. Pore space characteristics of sample INF-MB3 by µCT analysis. a) The packstone/grainstone sample lacks alteration. Scale: 0.5 mm. Plug diameter 13 mm. b) Vertical section shows minor porosity >26 µm. The dashed line marks the position of c. c) Reconstructed pore space. Colors mark parts of the pore system connected by throats >26 µm.

FIG S7. Number of bp and sequences before and after sequence trimming visualized as a combined box plot and violin plot. Statistical significance was tested using Wilcoxon signed-rank test.

FIG S8. Estimated coverage of metagenome data sets based on k-mer based redundancy using *nonpareil* [1,2]. The dashed red line indicates 95% coverage.

FIG S9. Assembly statistics for *megahit* [3] and *metaspades* [4] assemblies.

Fig S10. DNA fragment size distribution. Fragment sizes were deduced from 100k sampled read pairs mapped onto assembled contigs (> 1 kbp).

Fig S11. DNA damage pattern analysis of *Cand.* Rokubacteria contigs. Contigs were subsampled based on the taxonomic affiliation, which was determined with *kaiju* [5]. Quality-controlled sequence reads were mapped onto assembled contigs (> 1 kbp). The damage pattern analysis was carried out with *mapdamage* (v.2.2.1) [6]. The plots show the substitution frequency (5pCtoT, 3pGtoA) versus the relative position (from the 5p and 3p end). n = number of contigs > 1kbp considered for the analysis, cov = mean coverage of the contigs.

Table S1. Statistics sequence data processing. pwd = powdered sample, pc = rock pieces sample, QC = quality control.

Table S2. Identified contaminants for different taxonomic ranks. Freq = frequency, prev = prevalence, p.freq = tail probability at value R, p.prev = tail probability of the chi-square distribution for the respective taxon based on presence/absence in true samples and negative controls, p = p-value from Fisher's exact test, NA = not available. Please see [7] for details regarding the mentioned metrics.

Table S3. Phylum-level taxonomic profiles. lib_blk = library blank, ex_blank = extraction blank, pc = rock pieces sample, pwd = powdered sample.

Table S4. Basic assembly statistics and results from read recruitment.

Table S5. Phylum-level taxonomic profiles of assembled contigs (> 1 kbp).

Table S6. Taxonomy and quality information regarding recovered genome bins based on *checkm* [8] output.

Table S7: Functional profile based on KEGG Lvl3 Orthologies. Abundances are given as CoPM. CoPM = copies per million.

Table S8: Functional profile based on a subset of KEGG pathways. CoPM = copies per million, logCoPM = log copies per million.

Note: All supplementary tables are provided in one combined spreadsheet.

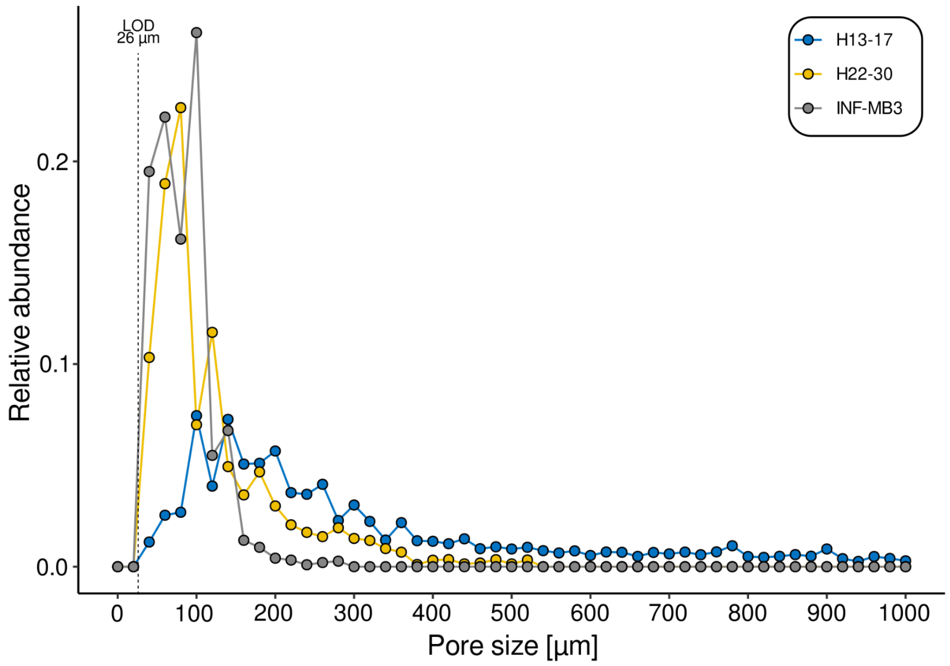
LOD
26 µm
H13-17
H22-30
INF-MB3
Relative abundance
0.0
0.1
0.2
0
100
200
300
400
500
600
700
800
900
1000
Pore size [µm]

Figure S1

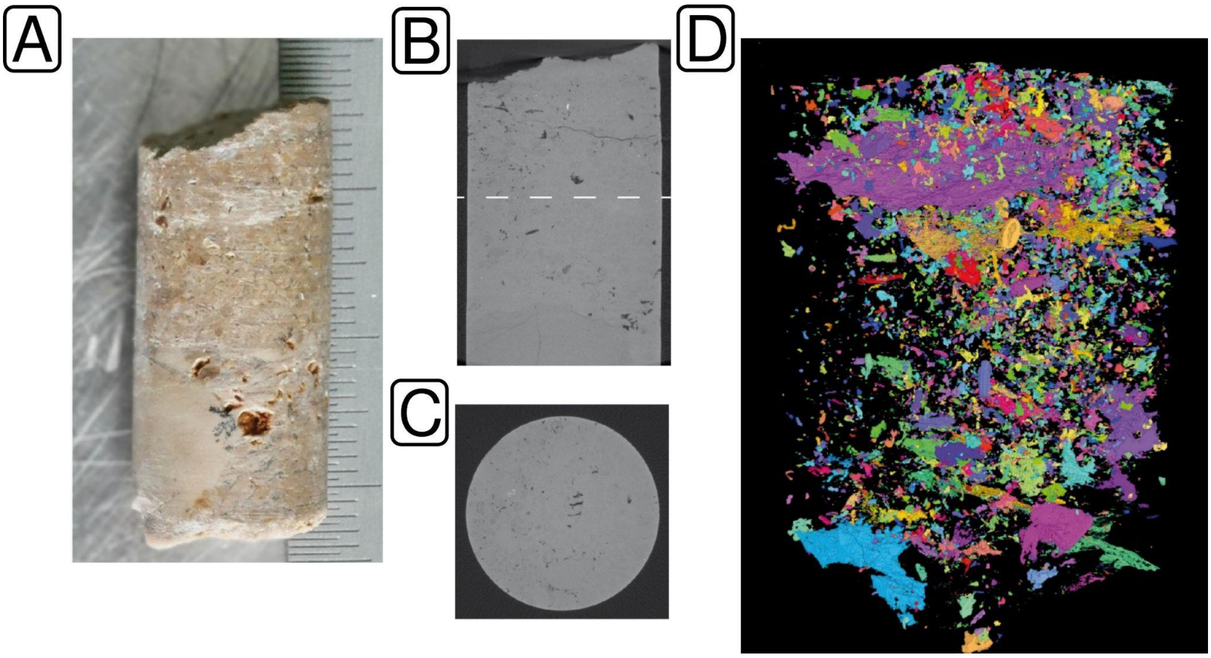

Figure S2

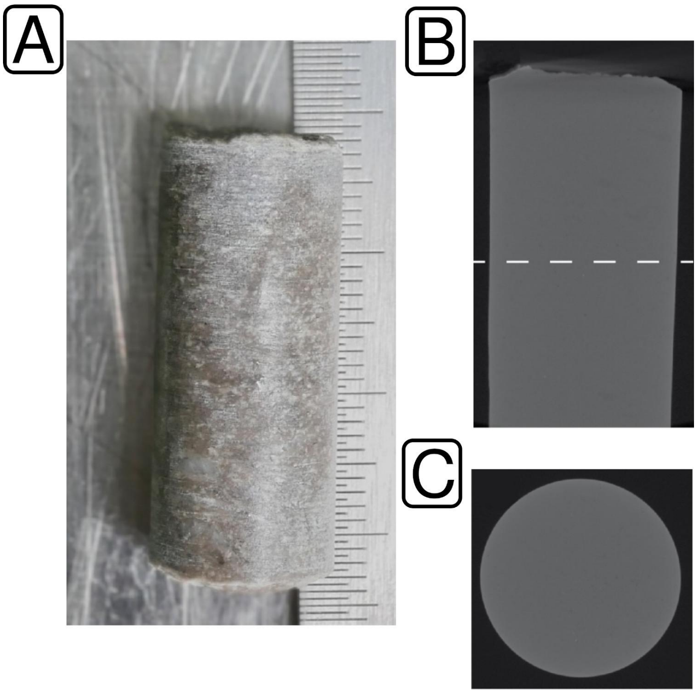

Figure S3

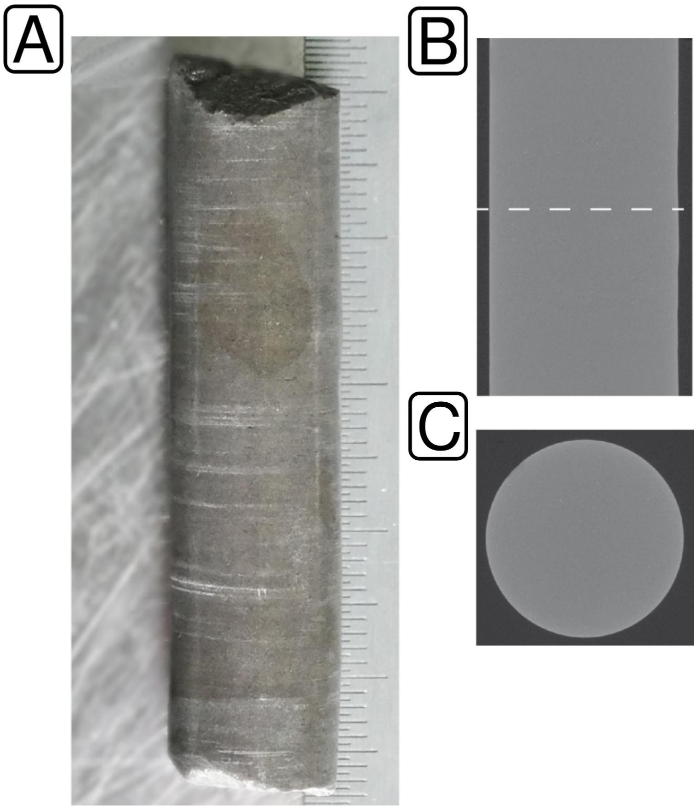

Figure S4

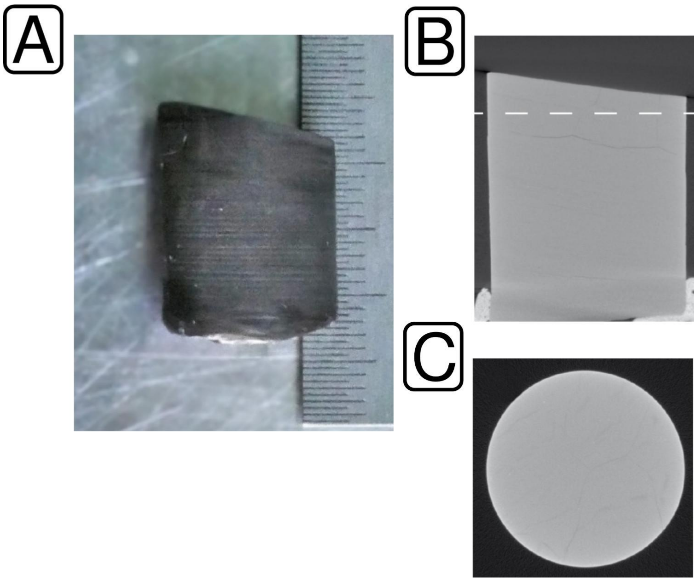

Figure S5

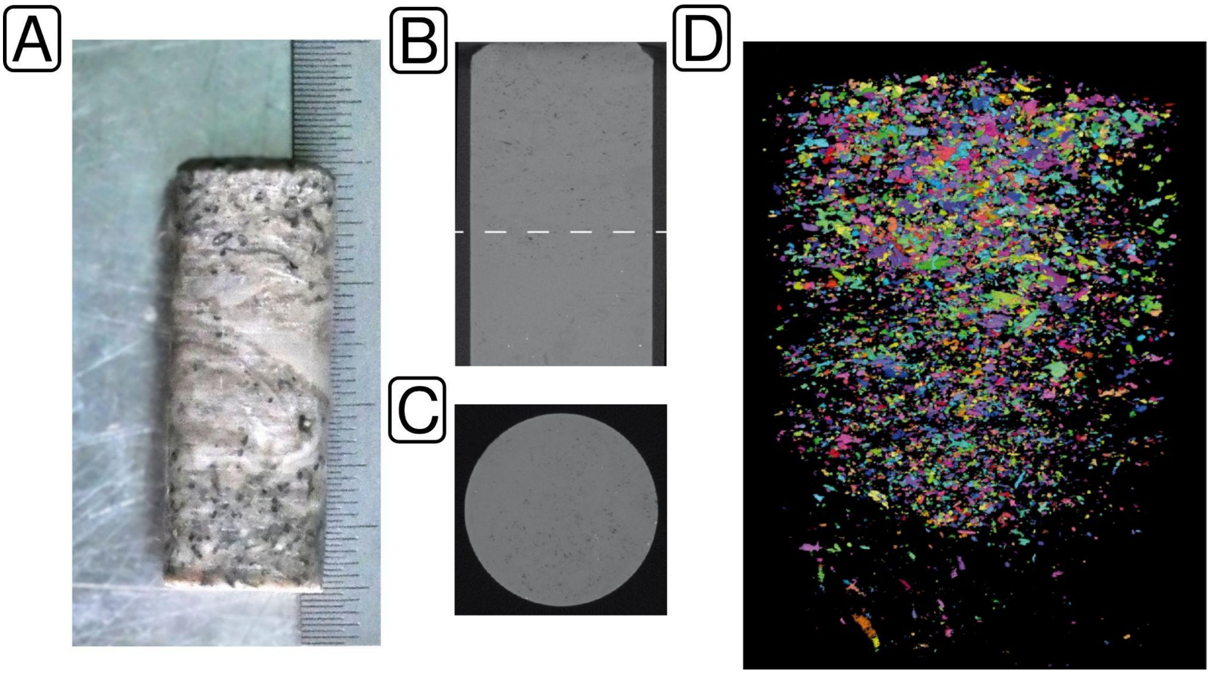

Figure S6

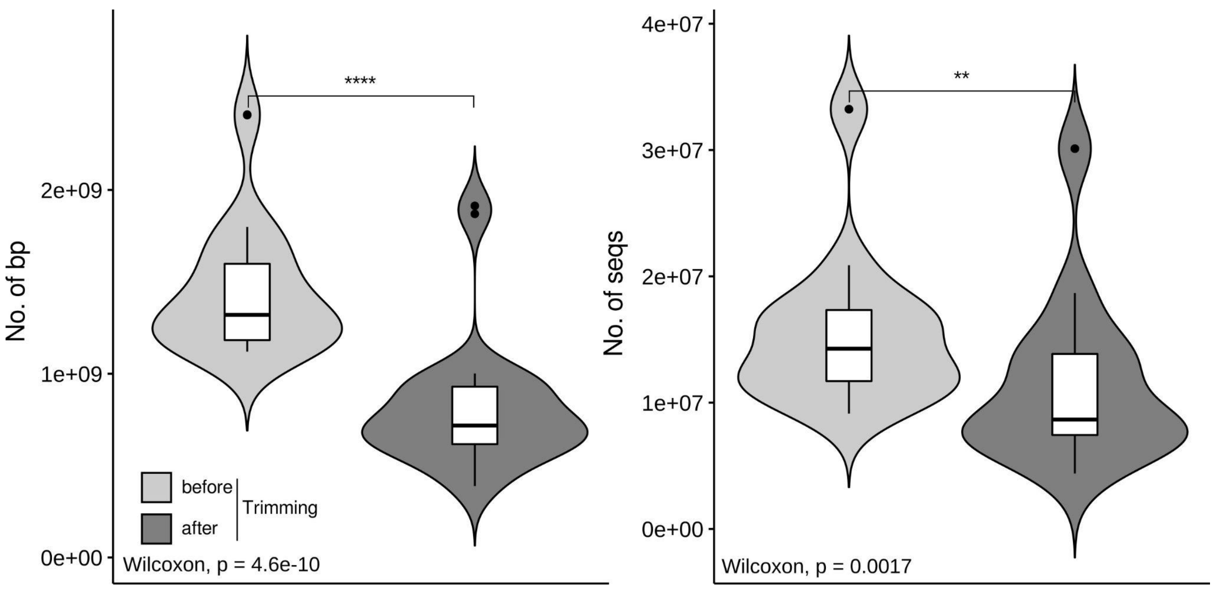
****
2e+09
1e+09
0e+00
No. of bp
before
after
Trimming
Wilcoxon, p = 4.6e-10
**
4e+07
3e+07
2e+07
1e+07
0e+00
No. of seqs
Wilcoxon, p = 0.0017

Figure S7

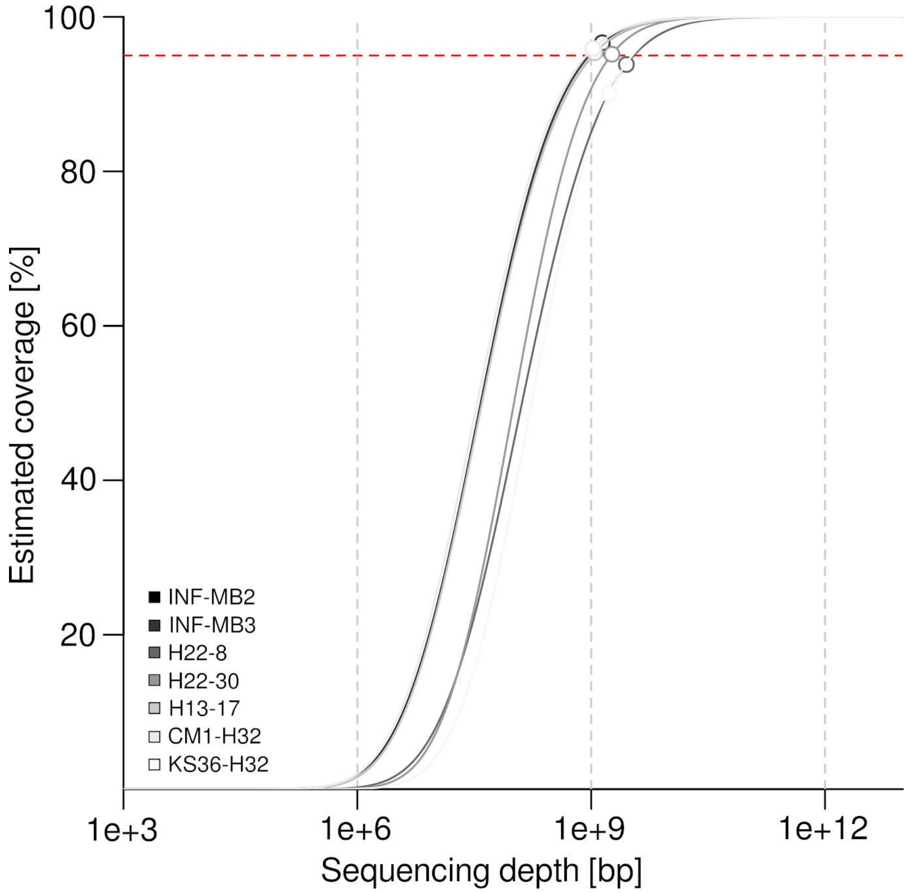
100
80
60
40
20
Estimated coverage [%]
INF-MB2
INF-MB3
H22-8
H22-30
H13-17
CM1-H32
KS36-H32
1e+3
1e+6
1e+9
1e+12
Sequencing depth [bp]

Figure S8

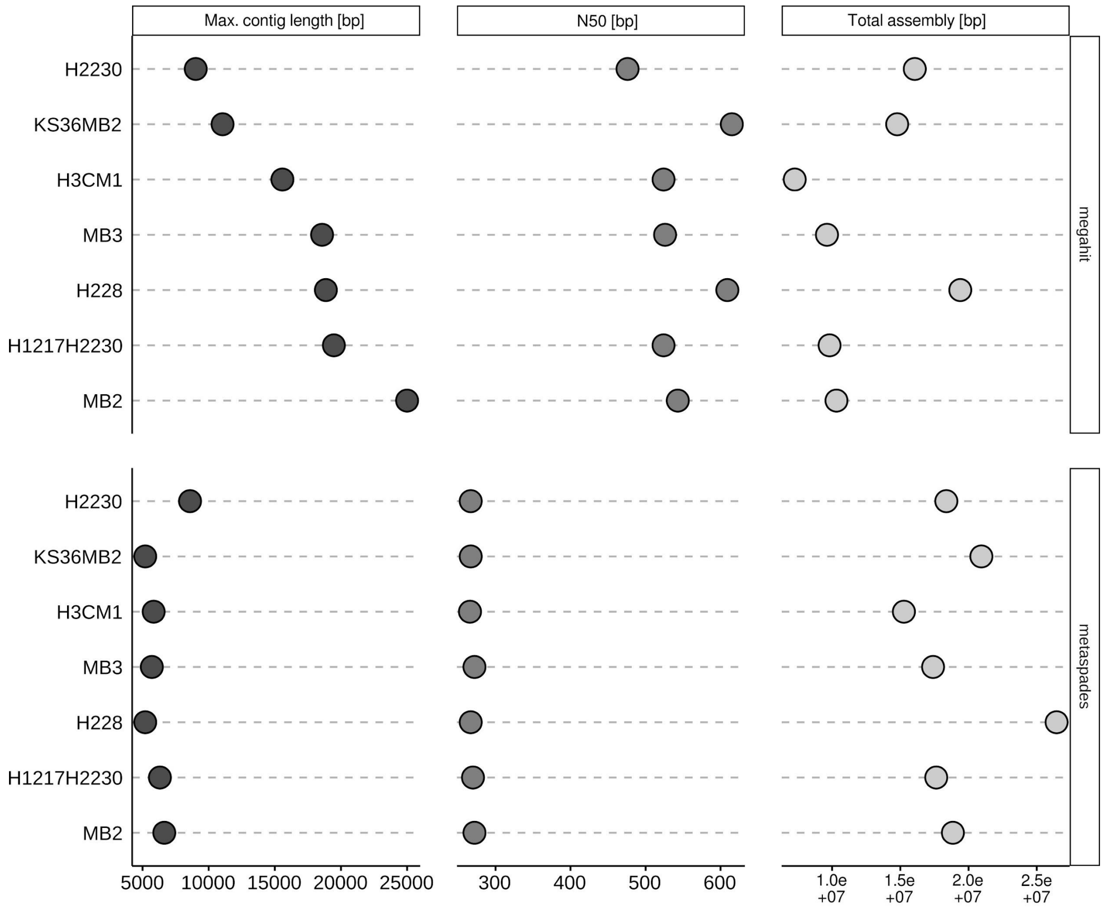
Max. contig length [bp]
N50 [bp]
Total assembly [bp]
megahit
metaspades
H2230
KS36MB2
H3CM1
MB3
H228
H1217H2230
MB2
5000
10000
15000
20000
25000
300
400
500
600
1.0e+07
1.5e+07
2.0e+07
2.5e+07

Figure S9

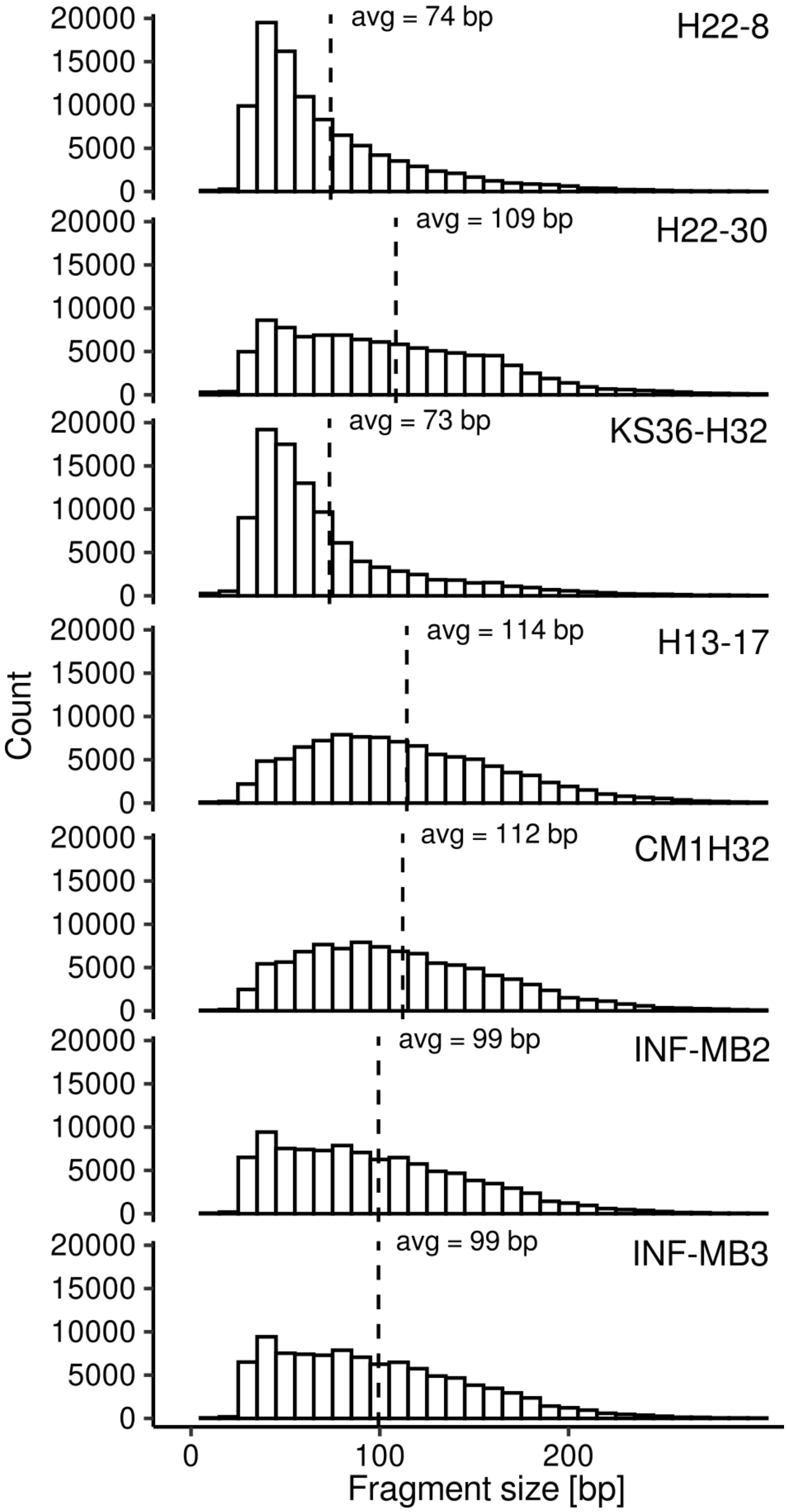
avg = 74 bp
H22-8
avg = 109 bp
H22-30
avg = 73 bp
KS36-H32
avg = 114 bp
H13-17
avg = 112 bp
CM1H32
avg = 99 bp
INF-MB2
avg = 99 bp
INF-MB3
20000
15000
10000
5000
0
Count
0
100
200
Fragment size [bp]

Figure S10

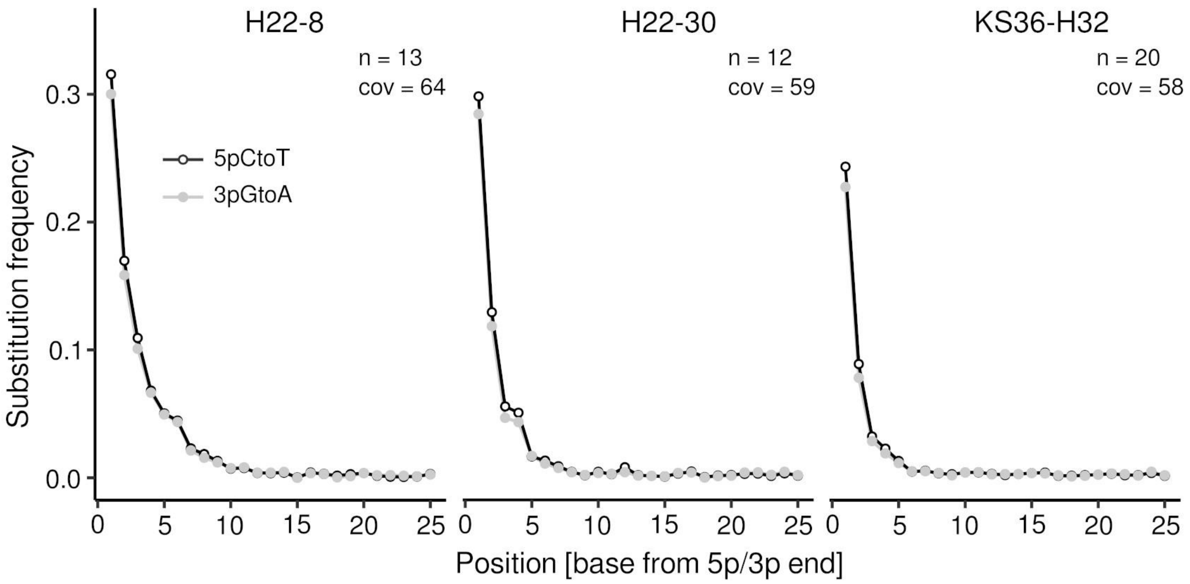
H22-8
n = 13
cov = 64
H22-30
n = 12
cov = 59
KS36-H32
n = 20
cov = 58
5pCtoT
3pGtoA
Substitution frequency
0.0
0.1
0.2
0.3
0
5
10
15
20
25
Position [base from 5p/3p end]

Figure S11

References

1. Rodriguez-R, L. M. & Konstantinidis, K. T. Nonpareil: a redundancy-based approach to assess the level of coverage in metagenomic datasets. *Bioinformatics* **30**, 629–635 (2014).
2. Rodriguez-R, L. M., Gunturu, S., Tiedje, J. M., Cole, J. R. & Konstantinidis, K. T. Nonpareil 3: Fast Estimation of Metagenomic Coverage and Sequence Diversity. *mSystems* **3**, (2018).
3. Li, D., Liu, C. M., Luo, R., Sadakane, K. & Lam, T. W. MEGAHIT: An ultra-fast single-node solution for large and complex metagenomics assembly via succinct de Bruijn graph. *Bioinformatics* **31**, 1674–1676 (2014).
4. Nurk, S., Meleshko, D. & Pevzner, P. {metaSPAdes}: a new versatile de novo metagenomics assembler. *Quantitative Biology* (2016).
5. Menzel, P., Ng, K. L. & Krogh, A. Fast and sensitive taxonomic classification for metagenomics with Kaiju. *Nat. Commun.* **7**, 1–9 (2016).
6. Jónsson, H., Ginolhac, A., Schubert, M., Johnson, P. L. F. & Orlando, L. mapDamage2.0: fast approximate Bayesian estimates of ancient DNA damage parameters. *Bioinformatics* **29**, 1682–1684 (2013).
7. Davis, N. M., Proctor, D. M., Holmes, S. P., Relman, D. A. & Callahan, B. J. Simple statistical identification and removal of contaminant sequences in marker-gene and metagenomics data. *Microbiome* **6**, 226 (2018).
8. Parks, D. H. *et al.* CheckM: assessing the quality of microbial genomes recovered from isolates, single cells, and metagenomes. *Genome Res.* **25**, 1043–1055 (2015).
